# Celebrating a decade of *JCI Insight* — reflections from a former Editor in Chief

**DOI:** 10.1172/jci.insight.202108

**Published:** 2026-01-09

**Authors:** Kathleen Collins

This year, 2026, marks an extraordinary milestone for *JCI Insight*: our tenth anniversary. I had the privilege to serve as the second Editor in Chief, from the fall of 2019 to the fall of 2024, and I write now — having passed the baton — both honored and deeply reflective about my tenure and the collaborative achievements that have shaped the Journal over this remarkable first decade.

## A journal’s evolution: 10 years of insight

*JCI Insight* was founded in January 2016 under the leadership of Howard A. Rockman, MD, of Duke University, with a vision to advance scientific discovery by emphasizing rigor, transparency, and accessibility. From its inception, the Journal set out to be not only a steward of the most robust clinical and translational research, but also a platform for new ideas and perspectives that challenge existing paradigms. Over the past ten years, we have evolved into a respected and innovative voice within the biomedical research community, serving as a venue for work that reshapes our understanding of disease mechanisms, propels medical innovation, and adapts to the shifting landscape of human health.

This growth could not have occurred without the dedication of a vibrant, diverse community of authors, reviewers, and readers whose collective expertise has given *JCI Insight* its unique character. Our core values —scientific excellence, thoughtful communication, and a deep commitment to open knowledge — have anchored us in these rapidly changing times. Even as external circumstances and scientific methods evolved, our mission has remained constant: promote high-impact, trustworthy, and accessible research.

## Steering through times of change (2019–2024)

The five years of my tenure as Editor-in-Chief coincided with an era of both unprecedented challenges and profound opportunities. When I began in the fall of 2019, I do not think any of us could have imagined the coming transformation in our professional and personal lives. By March 2020, the world stood stunned as the spread of COVID-19 upended the most basic norms in health care, science, and society at large. The pandemic quickly became a defining moment for so many — patients, providers, researchers, and families. We were all forced to confront urgent and deeply personal questions: Should schools, offices, and public spaces close? Were family gatherings too risky? What sacrifices were necessary to protect the most vulnerable among us?

In these moments, the responsibility of medical professionals and scientists was made even more apparent. We were tasked with empowering our patients and communities with the most accurate information available so that they could make truly informed decisions. In recent decades, our profession has rightfully shifted away from paternalism toward a prioritization of transparency, honesty, and patient-centered communication. This evolution is essential, not only to foster trust, but also to ensure that medical decisions reflect the values and goals of the most affected patients.

Likewise, at the population level, public health responses depend on transparent, consistent messaging. Without transparency, trust is eroded, misinformation flourishes, and collective action mechanisms break down. These lessons grew more salient over the course of the COVID-19 pandemic, as leaders and scientists worldwide struggled with how best to communicate critical information in an environment often clouded by uncertainty, fear, and competing interests.

*JCI Insight* took on a heightened responsibility during these years. While many journals sought to quickly disseminate COVID-19 research, we remained steadfast in upholding the standards that have always defined us: rigorous peer review, transparency in methodology and reporting, and a deep respect for the foundational principles of scientific trust. We mobilized to fast-track critical studies that could shape responses to the pandemic, while never sacrificing our commitment to robust evaluation and scientific rigor. I am deeply grateful to our Editorial Board, reviewers, and staff for their extraordinary dedication during this challenging period. Their work underscored, for me, the essential role of open-access publishing and the need for robust communication among scientists, clinicians, and the broader public.

## Expanding our vision: diversity, equity, and physician-scientist development

The COVID-19 years also thrust into the spotlight the persistent inequities within our health care and research systems. As Editor, I was especially aware of the opportunity — and obligation — we had to address these disparities in our work. While much work remains, I believe these efforts are foundational to sustaining the mission and relevance of *JCI Insight*.

An important pillar of the American Society for Clinical Investigation (ASCI) is its focus on supporting and sustaining the physician-scientist workforce across all career stages. For example, despite constituting only about 3% of medical school graduates, MD-PhDs account for nearly half of NIH-funded physician-scientists — a testament to their outsized impact at the intersection of scientific discovery and clinical care (1). Yet the workforce pipeline faces troubling signs: rigorous, lengthy training and persistent barriers deter many, especially those from underrepresented backgrounds. The result is a leaky pipeline that jeopardizes both the entry and advancement of new clinician-scientists, and ultimately, the future of our field.

A particular highlight of my tenure was the launch of the Physician-Scientist Development section of the journal — a dedicated category for studies and perspectives on innovative approaches to education, training, recruitment, retention, and advancement for physician-scientists at all career stages. We especially encourage submissions with robust metrics, data on outcomes, and actionable solutions. By creating a home for this type of research, we hope to foster creative dialogues and evidence-driven strategies to diversify and sustain this vital workforce for years ahead. I encourage all to explore the collection (https://insight.jci.org/collections/topic/physician-scientist-development) and contribute to this essential conversation.

## A community effort

None of these advances happened in isolation. The story of *JCI Insight*, and our ten-year milestone in particular, is a testament to the power of collaboration. The Journal is shaped and propelled forward by a community of authors, reviewers, editors, and engaged readers — each contributing to our shared commitment to excellence and impact. During my tenure, it was my privilege to work alongside an outstanding Editorial Board, dedicated staff, and inspiring society leaders, all imbued with a sense of purpose and mission.

## Looking forward with pride and purpose

Now, as a former Editor in Chief reflecting on a transformative chapter, I am filled with pride — and even more, with optimism for the future of *JCI Insight*. Significant challenges remain, both in science and across society, but there is equally great promise if we proceed with boldness, integrity, and collaboration. The Journal’s next decade will build on the strong foundation of its first ten years, as it continues to champion scientific rigor, open access, equity, and direct, honest communication — the indispensable pillars of trust in medicine and research.

To all who have contributed to *JCI Insight*’s remarkable story over the last decade: Thank you. It has been the greatest privilege of my career to help guide this Journal through times of adversity and extraordinary growth. I look forward — with excitement and confidence — to the discoveries, insights, and innovations that the future holds.

## Version 1 01/09/2026

Electronic publication
